# Engineering Three-Dimensional Moiré Flat Bands

**DOI:** 10.1021/acs.nanolett.1c01684

**Published:** 2021-09-13

**Authors:** Lede Xian, Ammon Fischer, Martin Claassen, Jin Zhang, Angel Rubio, Dante M. Kennes

**Affiliations:** † Songshan Lake Materials Laboratory, 523808 Dongguan, Guangdong China; ‡ Center for Free Electron Laser Science, Max Planck Institute for the Structure and Dynamics of Matter, 22761 Hamburg, Germany; § Institut für Theorie der Statistischen Physik, RWTH Aachen University and JARA-Fundamentals of Future Information Technology, 52056 Aachen, Germany; ∥ Department of Physics and Astronomy, University of Pennsylvania, Philadelphia, Pennsylvania 19104, United States; ⊥ Center for Computational Quantum Physics, Simons Foundation Flatiron Institute, New York, New York 10010 United States; # Nano-Bio Spectroscopy Group, Departamento de Fisica de Materiales, Universidad del País Vasco, UPV/EHU- 20018 San Sebastián, Spain

**Keywords:** Twisted moiré materials, Flat bands, Strongly correlated electrons, Superconductivity, Ab Initio calculations

## Abstract

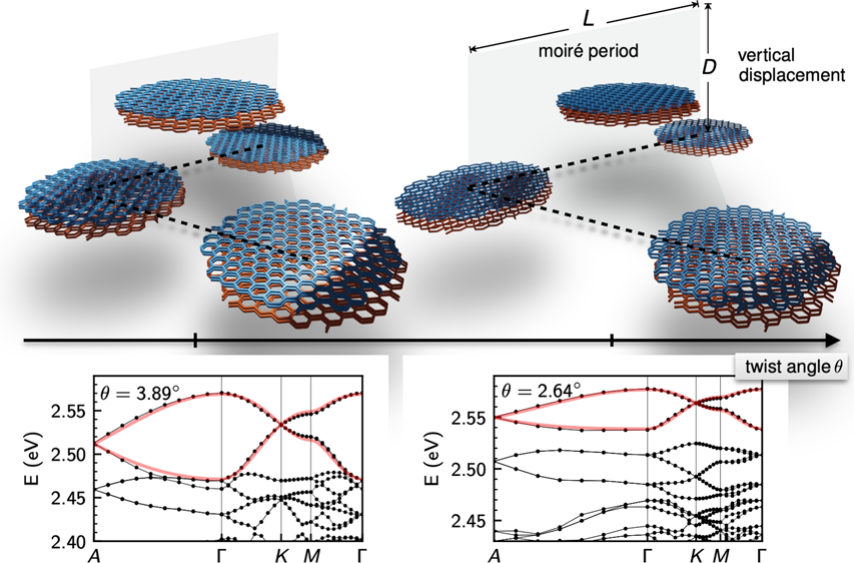

Twisting two adjacent layers of van der Waals materials with respect to each other can lead to flat two-dimensional electronic bands which enables a wealth of physical phenomena. Here, we generalize this concept of so-called moiré flat bands to engineer flat bands in all three spatial dimensions controlled by the twist angle. The basic concept is to stack the material such that the large spatial moiré interference patterns are spatially shifted from one twisted layer to the next. We exemplify the general concept by considering graphitic systems, boron nitride, and WSe_2_, but the approach is applicable to any two-dimensional van der Waals material. For hexagonal boron nitride, we develop an *ab initio* fitted tight binding model that captures the corresponding three-dimensional low-energy electronic structure. We outline that interesting three-dimensional correlated phases of matter can be induced and controlled following this route, including quantum magnets and unconventional superconducting states.

## I Introduction

In the past few years, twisting adjacent layers of van der Waals materials has emerged as a versatile route to control the ratio between kinetic, potential, and vibrational energy of two-dimensional systems. Central to the idea of twistronics, the selective suppression of kinetic energy scales permits tuning materials into a regime dominated by electronic interactions, as well as precise control over electronic filling via gating.^1,2^ Early experimental and theoretical studies concentrated on graphetic systems of different kinds, such as twisted bilayer graphene,^3−7^ twisted double bilayer graphene,^8−12^ trilayer rhombohedral graphene on hexagonal boron nitride,^13,14^ and twisted mono-bilayer graphene.^15,16^ More recently, twisted transition metal dichalcogenides (TMD) moved into the center of attention as another important class of van der Waals materials, that is, WSe_2_,^17−19^ MoS_2_,^20−22^ and TMD heterostructures,^23−26^ allowing access to new regimes beyond graphitic systems. With more of these phenomena within experimental reach, twisted van der Waals materials are increasingly viewed as potential avenues toward solid-state based platforms of quantum control and quantum materials with properties on demand.^2,27^ Furthermore, the twist angle allows one to control those systems to such a high degree that moiré-aided metrology outperforms the current gold standard regarding structural questions in van der Waals materials.^28^ One important question guiding theoretical and experimental research efforts concerns the exploration of the tremendous combinatoric space of chemical compositions of van der Waals materials to shed light onto the basic question of which additional phenomena might be accessible using twistronics. In the quickly expanding cosmos of twisted van der Waals materials, one guiding principle remains the control of the low-energy degrees of freedom, which might realize prototypical models of condensed matter research in a more rigid, clean, and penetrable context.^2^ In this spirit and in addition to the directions already outlined above, twisted hexagonal boron nitride was shown to harbor entire families of flat bands,^29,30^ and twisted two-dimensional magnets, such as CrI_3_, were identified to realize moiré skyrmion lattices and noncollinear twisted magnetic phases.^31,32^ Beyond realizing different phenomena in two-dimensions using twist as a control paradigm, one might ask whether entirely different dimensionalities can (effectively) be addressed. Twisted two-dimensional monolayers of monochalcogenides (e.g., GeSe) were shown to allow access to the one-dimensional limit with twist^33,34^ providing the same unprecedented level of control as in the two-dimensional counterparts.

However, a practical generalization of twistronics to three dimensions with suppressed kinetic energy scales remains an outstanding challenge (aside from the possibility of adding further synthetic dimensions^35,36^). Here, we address this by showing that stacking van der Waals materials in a predefined fashion allows one to engineer three-dimensional moiré flat bands. With this advance, the realization of three-dimensional systems controlled by twistronics is no longer elusive, completing the list of systems controlled by twistronics in one, two, and now three dimensions. Our idea works generically and relies mainly on basic geometric arguments. Importantly, it is achievable within recent experimental advances to fabricate bulklike artificial twisted materials^37−39^ and can be applied to any of the many van der Waals materials, which we will exemplify here for three important materials: graphitic systems, WSe_2_, and hexagonal boron nitride. The last of these will be examined in more details and we provide a full *ab initio* characterization for its three-dimensional twist-dependent band structures. We then consider the effects of correlations on the low-energy bands and find three-dimensional magnetic and superconducting states which can be realized as a function of twist angle.

## II Stacking Approaches

**Figure 1:**
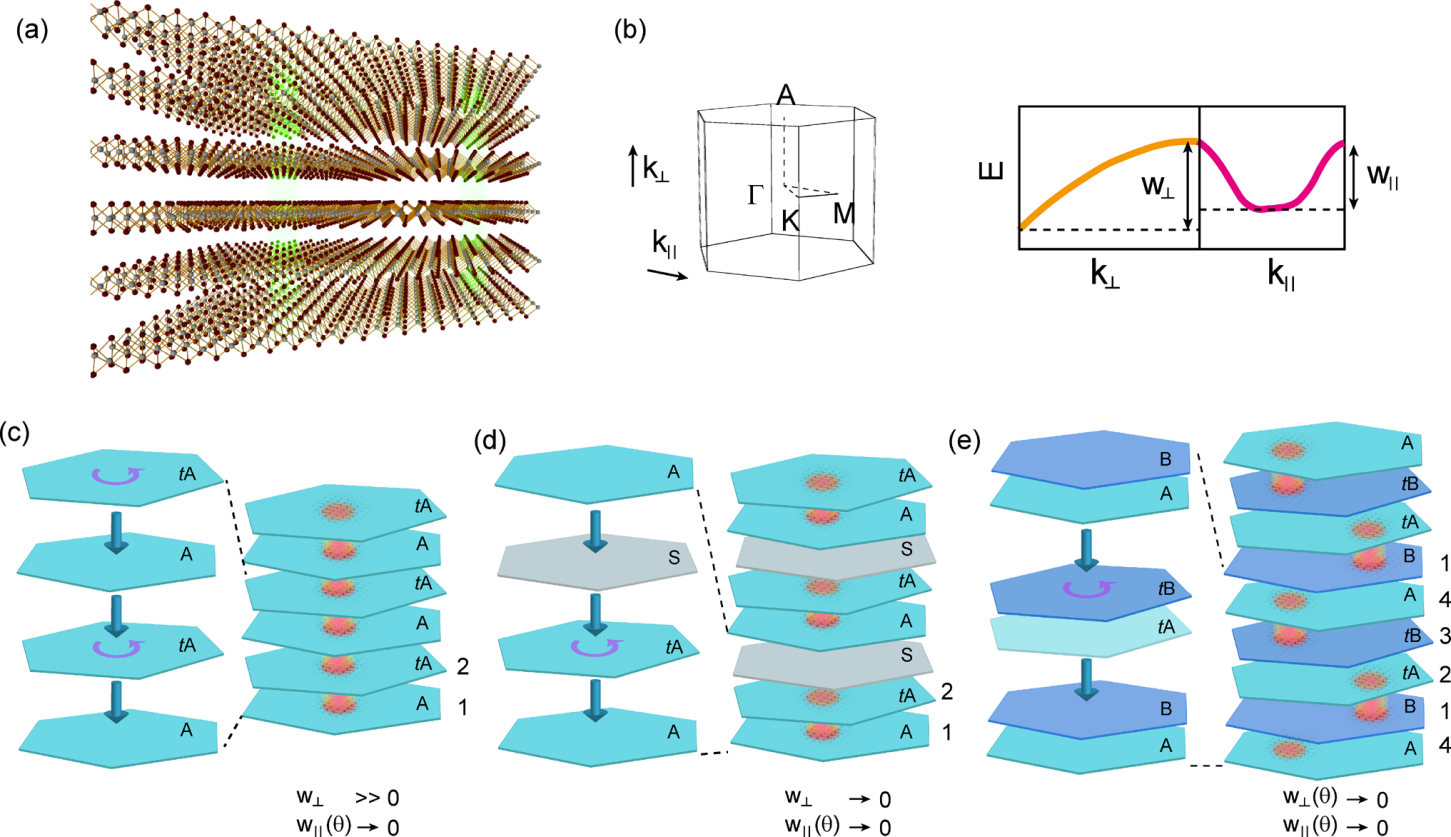
Stacking ideas to construct 3D flat bands. Panel a exemplifies the general idea of stacking up lattice sites defined by the moiré potential in a successive way to approach the three-dimensional limit. Panel b shows the Brillouin zone and defines the out-of plane *k*_⊥_ and in-plane *k*_∥_ directions (left) as well as a cartoon of the corresponding bandwidths w_⊥_ and w_∥_ along these directions (right). Panels c–e show three different configurations to create three-dimensional materials out of stacks of two-dimensional twisted van der Waals materials. The orange area indicates the moiré lattice sites where the charge density has localized. When these moiré lattice sites align atop of each other (c) the bands become flat in two of the three directions only. When using spacer layers (d) the bandwidth can be reduced in all three dimensions, but the bandwidth along the stacking direction cannot be controlled by the twist angle. If moiré lattice sites by geometric reasons (see main text) are shifted from layer to layer (e), three-dimensional flat bands with the flatness in all three dimensions being controlled by the twist angle emerge.

The general idea shown in Figure 1a relies on successively stacking up lattice sites defined by the moiré potential. In Figure 1b, we define the Brillouin zone, the in-plane and out-of-plane directions *k*_⊥_ and *k*_∥_, as well as a cartoon of the corresponding band widths used in the following discussion. We are looking for ideal 3D flat bands that satisfy the following conditions: (1) the bandwidth is controllable by twist angles in all three dimensions and (2) the system is periodic along all three dimensions such that it remains a well-defined crystal. Therefore, our work is distinct from previous works that study intrinsic 3D flat bands in some solids^40−42^ with a fixed bandwidth and little tunability. Our approach also is distinctly different to proposals such as the 3D chiral twisted structure^43,44^ that renders the system a quasicrystal. To this end, we start by considering three different stacking patterns illustrated in Figure 1c–e.

First, we consider stacking monolayers of van der Waals materials in an alternating fashion as depicted in Figure 1c, meaning that every second layer is aligned perfectly while adjacent layers have a relative twist angle between them. This can be regarded as stacking twisted bilayers repeatedly. Following this route, moiré patterns form by the interference between adjacent layers. This type of stacking has been intensively investigated for the study of two-dimensional flat bands in twisted trilayer and multilayer graphene.^45,46^ Viewed top-down, the moiré lattice sites, where in-plane charge density localizes, align on top of each other. As a consequence, the electronic bands become flat within the plane just as is the case for two twisted sheets of van der Waals materials. Conversely, the alignment of moiré regions along the out-of-plane direction retains substantial band dispersion in this direction due to a significant amount of hybridization between moiré sites at adjacent layers (see Figure S1 in the SI for an example of 3D twisted boron nitride with such stacking). While this allows one to effectively engineer quasi-one-dimensional systems with very low residual coupling along the remaining two spatial directions, that is, an interesting opportunity of material engineering in its own rights (for a similar quasi-one-dimensional system it was shown that in-plane confinement albeit imposed by an magnetic field gives rise to a 3D quantum Hall effect^47^), it does not allow one to realize three-dimensional moiré flat bands.

Second, one might consider the case in which twisted van der Waals materials with flat bands in their two spatial directions are stacked on top of each other with an insulating buffer layer in between as shown in panel Figure 1d. The properties and thickness of the buffer-layer could then be adjusted such that the hopping from one twisted sheet of van der Waals materials to the next sheet is suppressed substantially. This would lead to flat electronic bands in all three spatial dimensions. However, such an approach has multiple problems. First, the flatness of the bands in the out-of-plane direction is mainly determined by the residual coupling between neighboring moiré charge localization sites across the insulating layers. This limits available band structures that can be engineered quite substantially compared to the flexible control that twist angle offers with respect to the remaining two directions. Second, with a buffer layer there is no guarantee to keep the moiré sites across the buffer layer well aligned, that is, the centers of the moiré sites of neighboring twisted pairs can relocate to different in-plane positions when stacking up. This could introduce a significant amount of disorder along the out-of-plane direction such that the system is no longer a well-defined crystal.

Third, to remedy the shortcomings of the previous two stacking approaches we present an idea using a stacking sequence in which the moiré charge localization sites simply due to geometric considerations do not form atop of each other but are shifted with respect to the out-of-plane directions of the van der Waals materials used. Such a configuration can be constructed by expanding the basic stacking unit, for example, from a twisted bilayer to a twisted double bilayer. This is visualized in Figure 1e. Compared to the first approach, the repeating unit along the out-of-plane direction is changed from layers 1,2 in Figure 1a to layers 1–4 in Figure 1c. In this approach, layers 2,3 and layers 4,1 remain at their intrinsic Bernal AB stacking or AA′ stacking sequence as in the pristine bulk material and the twisting takes place only between layers 1 and 2 as well as 3 and 4 in the notation of Figure 1c. Although the in-plane crystal axis of layer 2 is aligned with those of layer 3, the atomic positions of the two layers are translated with respect to each other (as in intrinsic AB stacking), or flipped (as in intrinsic AA′ stacking). The same happens for layers 4 and 1. This naturally displaces the moiré charge localization sites in these layers with respect to each other, which are now separated by the moiré length scale. As the twist angle is decreased and the in-plane distance between moiré sites increases, so does the distance between sites on adjacent bilayers. Therefore, the idea of using natural (or intrinsic) bilayer as a stacking unit to construct alternating patterns allows one to engineer robust flat bands in all three dimensions with the flatness being continuously controlled by the twist. Thus, it satisfies the condition (1) we set for a ideal flat band system. Moreover, as we will show below, such an approach will also generate local stacking regions that resemble the stacking sequence in the pristine bulk crystal, which can act as a low-energy stabilization center to prevent disorder along the out-of-plane direction. Therefore, this approach also meets condition (2) of a nearly ideal robust flat band crystallographic system.

## III Flat Bands and Effective Low-Energy Model

**Figure 2:**
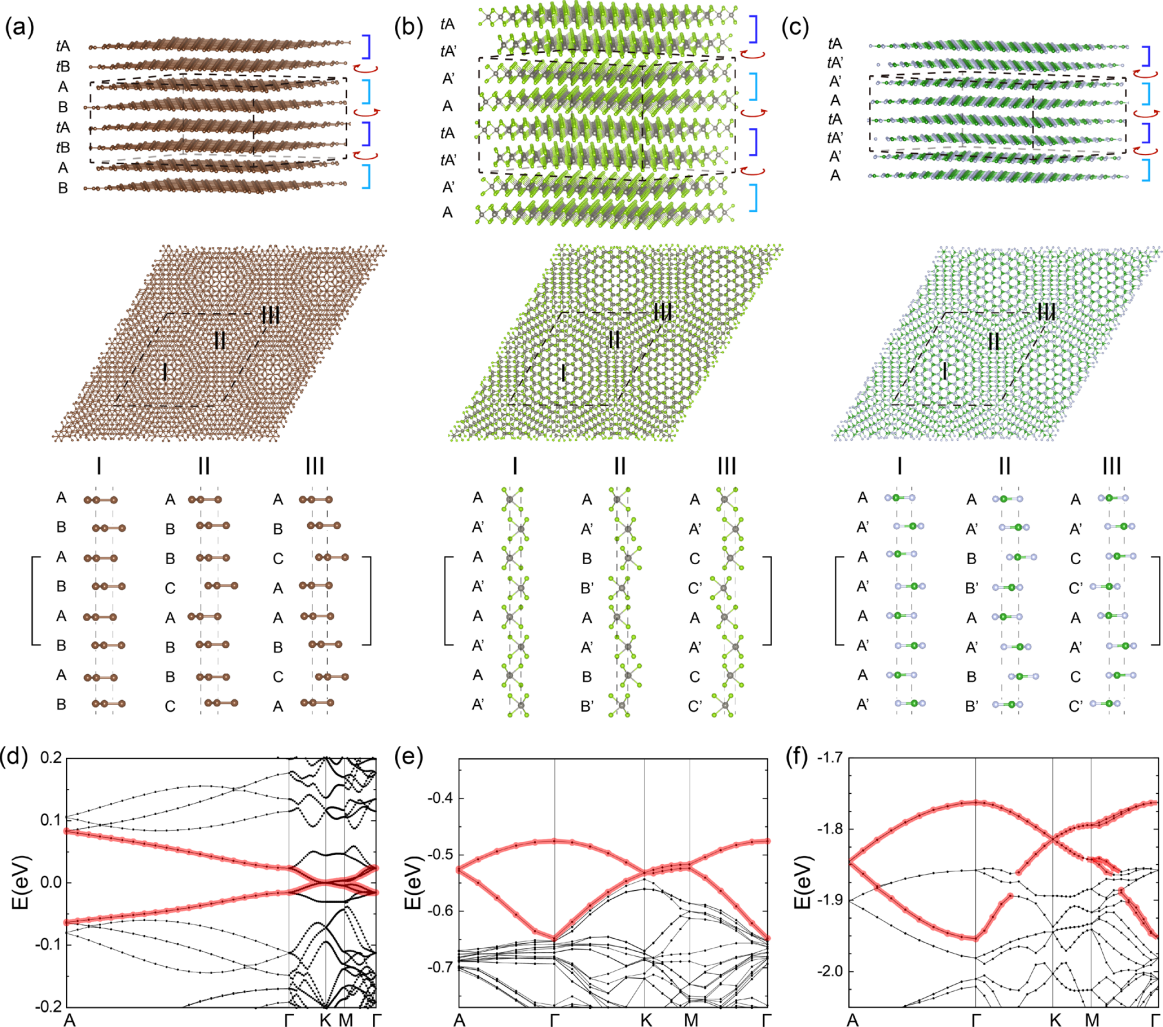
Three-dimensional flat bands for different materials, graphene, WSe_2_ and boron nitride. Atomic structures of 3D twisted graphene (a), WSe_2_ (b), boron nitride (c). The top and the middle panels show the perspective and the top views of the structures, respectively. The unit cells are indicated with dashed lines. The bottom panels show the local stacking sequence in the region I, II, and III indicated in the middle panels. The repeating units along the out-of-plane direction are indicated with solid brackets. (d–f) The corresponding band structures for graphene at 1.3° (d), for WSe_2_ at 5.08° (e), and for boron nitride at 5.08° (f). For smaller angles, the bands become increasingly flat and detach from other bands.

We put this very general idea to the test by first performing *ab initio* and tight-binding based characterizations of such stacked materials using bilayers of graphene, WSe_2_, as well as hexagonal boron nitride. All of these materials were successfully studied in the past for the twisted single bilayer case rendering them ideal starting points to explore the idea we put forward here. The results are summarized for a twist angle of 1.3° for graphene and 5.08° for both WSe_2_ and boron nitride in Figure 2. We show side and top views of the real space stacking in Figure 2a–c for graphene, WSe_2_, and boron nitride, respectively. The unit cell for these bulk twisted systems is formed by a twisted double bilayer as highlighted by dashed lines in the top row and the solid brackets in the third row. The bottom panels of Figure 2a–c show the local stacking sequence in the three representative regions shown in the middle row panels. The stacking sequence in region I is exactly the same as that in the intrinsic untwisted bulk crystal (AB stacking as in graphite, AA′ stacking as in 2H WSe_2_ and bulk boron nitride). This region is expected to pin the in-plane alignment of the layers and naturally prevent accidental layer displacements similar to what is discussed for the case of twisted trilayer graphene.^46^ In Figure 2d–f, we show the respective band structures. Since the angle is not very small for twisted WSe_2_ and twisted boron nitride, the bands still show substantial dispersion and the set of bands that is flattening, marked in red in Figure 2, has not fully separated from other bands yet (a more detailed study of decreasing the twist angle further is given below). The important feature to note is the width of the red set of bands with respect to variations of both the out-of-plane *k*_⊥_ (*A* → Γ path in the Brillouin zone) and in-plane *k*_∥_ (Γ → *K* → *M* → Γ path in the Brillouin zone) momentum. Both of these band widths in- and out-of-plane, called *W*_∥_ and *W*_⊥_ respectively, decrease as the twist angle approaches smaller values (see below and SI). Eventually these bands separate from the rest giving rise to perfectly isolated flat bands with comparable kinetic energy scales in all three spatial dimensions, whose magnitude can be tuned by the twist, as demonstrated next.

**Figure 3:**
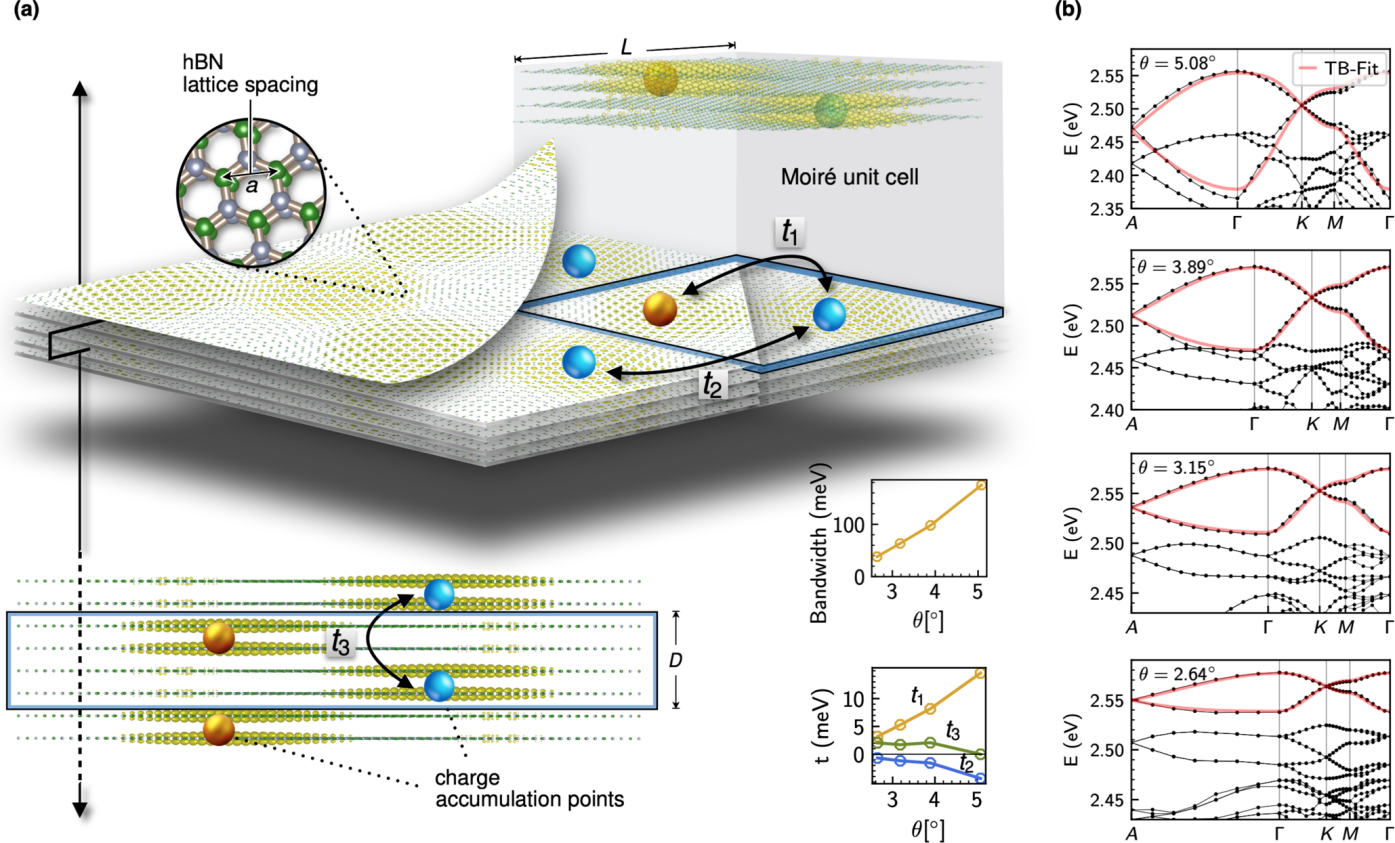
Low-energy model of three-dimensional twisted boron nitride (thBN). (a) Stacking pattern and moiré unit cell of thBN for θ = 5.08°. Emerging charge accumulation points (olive green) form an effective lattice that resembles AA-stacked hexagonal multilayers, where one site is shifted by *D*/2 in the *z*-direction (blue and gold spheres). (b) Results from our *ab initio* fitted tight-binding (TB) approach (see Supporting Information Section II for details) for different twist angles θ, taking up to next-nearest neighbor inter- and intralayer hopping terms *t*_1_,..,*t*_3_ into account. The bandwidth decreases continuously with the twist angle and eventually the low-energy bands detach from the rest of the spectrum.

We stress that the idea we present here is general and allows three-dimensional flat band engineering also in other materials even beyond the ones discussed explicitly above. However, we are going to illustrate the relevance of this new concept to electronic band engineering taking the specific case of hBN (see Figure S2 in the SI for the case of WSe_2_). The choice of boron nitride is made for convenience (and for being widely used as protective 2D material), as the absence of sharp magic angles, makes it particularly feasible to large scale numerics providing a full-fledged *ab initio* characterization of the material’s band structure in three dimensions. As the relaxation of twisted boron nitride does not significantly alter the band structure according to the previous work,^29^ we fix the atomic structure in the large-scale *ab initio* calculations. Our results are summarized in Figure 3. In panel Figure 3a, we show top and side views of the charge localization within the moiré unit cell and the position of the B and N atoms. We choose intrinsic AA′ bilayers (as in pristine bulk hBN) as the building blocks of our three-dimensional structure and therefore there are no ferroelectric domains as recently reported for twisted bilayer systems.^48,49^ Generalizing this constitutes an intriguing avenue of future research. In Figure 3b, we summarize the *ab initio* band structure obtained for different twist angles. Importantly, as we approach smaller values of the twist angle, both the in-plane and out-of-plane bandwidth *W*_∥_ and *W*_⊥_, which are the same with such stacking, decrease and the flat bands detach from the other bands in the spectrum. Strong charge localization marked by blue and golden spheres in Figure 3a highlight the emergence of a corresponding three-dimensional effective low-energy tight-binding model (see Supporting Information Section II for details). The model we obtain for the case of boron nitride is an in-plane triangular lattice stacked out-of-plane, such that the lattice sites of one plane reside in the center of the triangular lattice of the next plane.

The success of fitting the flat bands within such a low-energy tight-binding model including only short ranged hoppings *t*_1_, *t*_2_, and *t*_3_ on the moiré scale as denoted in Figure 3a is demonstrated in Figure 3b. By fitting the three hopping parameters to the full *ab initio* band structure for different angles, almost perfect agreement is achieved (consistent with the earlier study of a single twisted hBN bilayer^29^). The smaller panels left to Figure 3b show the extracted values of the hopping as well as the overall bandwidth (in this case *W*_⊥_ = *W*_∥_), demonstrating the success of three-dimensional flat band engineering by the twist proposed here. In particular, the hopping parameters *t*_1..3_ of the low-energy model prove that our initial claim of full twist angle control holds in the case of twisted hBN: the interlayer hopping *t*_3_ is nearly independent of the twist angle (small deviations occur for larger twist angles due to mixing of low-energy and valence bands, Figure 3b), whereas the in-plane hopping *t*_2_ and the mixed inter/intralayer term *t*_1_ decrease continuously. Such an effective low-energy model is immensely useful as it can be treated much more efficiently. As a direction of future research, and building on the above results, one should set up a continuum theory to further analyze the emergence of three-dimensional flat bands.

## IV Correlated Phases of Matter

**Figure 4:**
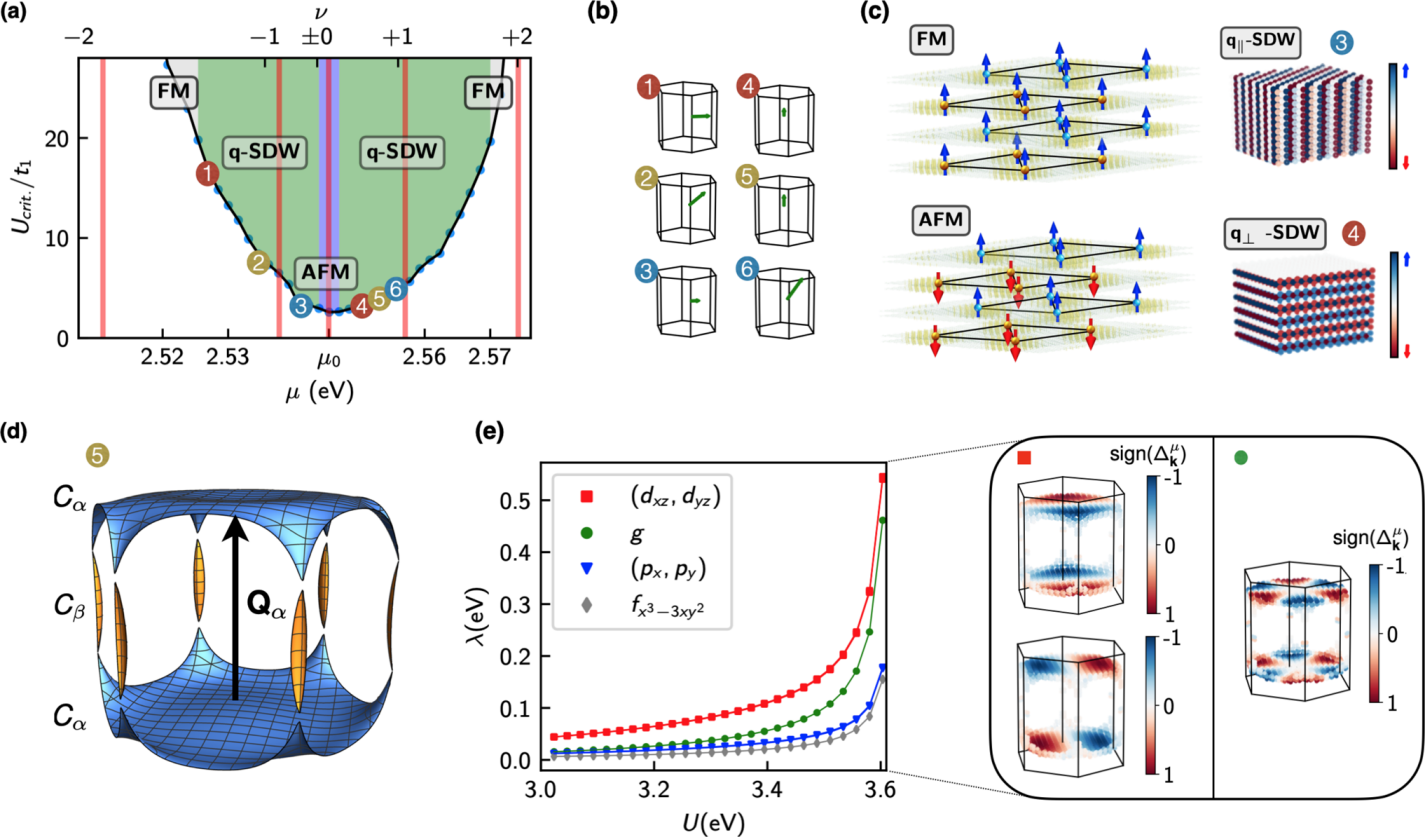
Correlated phases in three-dimensional twisted hexagonal boron nitride for twist angle θ = 3.15°. (a–c) The RPA analysis reveals a variety of magnetic states including AFM at charge neutrality, FM order for strong electron/hole doping as well as general spin-density waves q-SDW with periodic patterns in all three spatial dimensions. (d) For μ = μ_0_ + 5 meV, the Fermi surface of thBN is almost perfectly nested along the vector **Q**_α_ resulting in a strong enhancement of particle–particle scattering between these sheets. In particular, the preferred superconducting gap symmetry (e) is 2-fold degenerate and of type (d_*xz*_, d_*yz*_). Below *T*_c_, the system will minimize its Ginzburg–Landau free energy by assuming the chiral linear combination (d_*xz*_ ± *i*d_*yz*_) and thus the gap parameter breaks time-reversal symmetry.

We employ the effective low-energy tight-binding model to outline a putative unexplored phase diagram that could be accessible via the three-dimensional twistronics approach. To this end, we consider a local Hubbard interaction *U* added to the effective flat band model for θ = 3.15° as discussed above. We note that a more realistic model should include longer ranged interactions as well, which should be characterized from first principles. Such a study is unfortunately beyond the scope of the present work and most likely requires a fundamental methodological advance to treat the huge three-dimensional unit cell (containing many tens of thousands of atoms at small twist angles). Here, however, we provide the first step along a characterization of elusive and exciting correlation effects and aim to identify interesting states of matter already present at the level of a Hubbard interaction. To achieve this, we first perform a random phase approximation (RPA) study of the system (see Supporting Information Section III) and identify a plethora of magnetic instabilities. A putative magnetic phase diagram is summarized in Figure 4a. As expected, we find ferromagnetic (FM) ordering tendencies as the flat bands are either filled or empty, albeit with a rather large critical *U*_crit_ driving the transition. Because of the bipartiteness of the lattice (sublattices A and B being charge localization sites marked by blue and golden spheres in Figure 3a), we find antiferromagnetic (AFM) ordering at half filling. In between these two phases a more general spin density wave with filling dependent ordering vector *q* emerges. *q* is illustrated in Figure 4b, while Figure 4c illustrates the magnetic ordering in real space for four different examples depending on the filling: FM, AFM, or a spin density wave with a wave vector lying either in the in-plane (*q*_∥_) or out-of-plane (*q*_⊥_) direction. We note that the ordering vectors in general do not align with the crystal axes and therefore, although the underlying mechanisms that determine ordered states (such as Fermi surface nesting or van Hove singularities) are analogous to the two-dimensional case, the phases we find here cannot be described in terms of quasi two-dimensional planes.

For interaction values *U* < *U*_crit_, there is no magnetic ordering and the system is paramagnetic. In this regime, spin and charge fluctuations may provide an effective pairing glue between the electrons leading to the formation of Cooper pairs. To pin down the pairing instability mediated by spin and charge fluctuations, we take the RPA corrected interaction vertex in the fluctuation exchange approximation (see Supporting Information Section III) and linearize the superconducting gap equation around the critical temperature *T*_*c*_ for slight electron doping μ = μ_0_ + 5 meV around half filling of the three-dimensional flat bands μ_0_ = 2.546 eV. In this scenario, only scattering events between Cooper pairs in the vicinity of the Fermi surface sheets *C*_α,β_, Figure 4d, contribute notably to the formation of a superconducting state with order parameter Δ_**k**_^μ^. The fact that (i) the Fermi surface sheets *C*_α_ are (almost) perfectly connected by the nesting vector **Q**_α_ at which particle–particle scattering is strongest and (ii) the effective pairing glue contained in the spin-singlet channel is purely repulsive for all scattering events, electron–electron pairing is conditioned on a relative sign change between the pairing form factors μ connected by the vector **Q**_α_, that is, . The linearized gap equation may be written as an eigenvalue problem where the eigenfunctions Δ_k_^μ^ corresponding to the largest eigenvalue λ yield the symmetry of the most prominent superconducting state, Figure 4e. Our calculations reveal that the leading gap parameter is of spin-singlet type and is 2-fold degenerate with symmetry classification (d_*xz*_, d_*yz*_). The two d-wave solutions are characterized by a nodal line along the *k*_*x*_- and *k*_*y*_-direction of the Brillouin zone and thus the system will minimize its Ginzburg–Landau free energy *F* below *T*_c_ by assuming the chiral linear combination (d_*xz*_ ± *i*d_*yz*_) (see Supporting Information Section III) which breaks time-reversal symmetry.

In conclusion, our work generalizes the idea of two-dimensional twistronics to the three-dimensional realm. The main notion relies on cleverly stacking adjacent layers in such a way that the hopping between adjacent charge puddles in all three dimensions gets successively suppressed as the twist angle is lowered. We argue that the proposed stacking method is robust toward small twist angle imperfections and inhomogeneities which might vary within one plane or between adjacent layers. Even more so, since our construction relies purely on geometric arguments even in the presence of imperfections these would simply reflect in slightly inhomogeneous hoppings, like they are present in any (not perfectly clean) crystal and which do not change the overall physics significantly. On the contrary, controlled variations of the twist angle might allow one to control the effective disorder and therefore provide a long sought after inroad into tunable strongly disordered systems from a condensed matter perspective. With this, we add the three-dimensional realm to the list of low-energy models that can effectively be realized in moiré structures. As a side product, we also provide an alternative of engineering effectively quasi-one-dimensional structures by placing the moiré sites on top of each other (first scenario in Figure 1). This is not at the center of attention in this work but allows one to access similar physics as discussed in the context of the quantum Hall effect in ref (47). We already reported on the rich behavior of correlation driven phases in engineered three-dimensional flat bands above, but another intriguing avenue of future research should also address the question of three-dimensional flat band engineering for purposes of controlling topological properties. This provides a very rich playground that directly opens up by our approach and the future will tell which topological phenomena, such as Weyl physics, and correlated phases beyond the ones discussed here might be tunable by three-dimensional twistronics.

## Supporting Information Available

Further details on *ab initio* calculations, extended data for low-energy tight-binding models, theoretical details of multiorbital random-phase approximation (RPA), and linearized gap equation (PDF)
