# Supplementary material for: Engineering Three-Dimensional Moiré Flat Bands
Source: Nano Lett. 2021 Sep 13;21(18):7519–26. doi: 10.1021/acs.nanolett.1c01684 (PMC8461648; doi:10.1021/acs.nanolett.1c01684)
Supplement: Supplementary file 1 — nl1c01684_si_001.pdf [file nl1c01684_si_001.pdf]

Supplemental Material for
Engineering Three Dimensional Moiré Flat Bands

Lede Xian,[1,2,†] Ammon Fischer,[3,†] Martin Claassen,[4] Jin Zhang,[2] Angel Rubio,[2,5,6,*] and Dante M. Kennes[3,2,**]

[1]*Songshan Lake Materials Laboratory, 523808 Dongguan, Guangdong, China*

[2]*Max Planck Institute for the Structure and Dynamics of Matter, Center for Free Electron Laser Science, 22761 Hamburg, Germany*

[3]*Institut für Theorie der Statistischen Physik, RWTH Aachen University and JARA-Fundamentals of Future Information Technology, 52056 Aachen, Germany*

[4]*Department of Physics and Astronomy, University of Pennsylvania, Philadelphia, PA 19104, USA*

[5]*Center for Computational Quantum Physics, Simons Foundation Flatiron Institute, New York, NY 10010 USA*

[6]*Nano-Bio Spectroscopy Group, Departamento de Fisica de Materiales, Universidad del País Vasco, UPV/EHU- 20018 San Sebastián, Spain*

[†] L.X. and A.F contributed equally to this paper.

[*] Corresponding author: angel.rubio@mpsd.mpg.de

[**] Corresponding author: dante.kennes@rwth-aachen.de

S I. COMPUTATIONAL DETAILS FOR 3D TWISTED GRAPHENE, WSE2 AND BORON NITRIDE

For the calculations of 3D twisted graphene, we construct the unit cell with a twisted double bilayer graphene at twist angles close to 0 degree, and impose periodic boundary condition along all three dimensions. As it is not realistic to optimize such a large system with density functional theory (DFT) calculations, we fix the lattice constant along the out-of-plane direction to be 13.415 Å, and set the in-plane lattice constant according to the twist angles such that it corresponds to 2.46 Å for a 1x1 cell. The atomic structure is relaxed using the LAMMPS code [1] with the same parameters as described in [2]. The intralayer interactions within each graphene layer are modeled via the second-generation reactive empirical bondorder (REBO) potential [3]. The interlayer interactions are modeled via the Kolmogorov-Crespi (KC) potential [4], using the recent parametrization of [5]. The relaxation is performed using the fast inertial relaxation engine (FIRE) algorithm [6].

We calculate the band structures for 3D twisted graphene using the tight-binding parametrization proposed in Ref. [7]

$$H_0 = \sum_{i,j} t(\mathbf{r}_i - \mathbf{r}_j) c_i^\dagger c_j. \tag{1}$$

Here, the operator $c_i^{(\dagger)}$ annihilates (creates) an electron in the p_z orbital of the carbon atom at site $\mathbf{r}_i$. The p_z electrons are coupled via Slater-Koster hopping parameters $t_{ij} = t(\mathbf{r}_i - \mathbf{r}_j)$

$$\begin{aligned} t(\mathbf{d}) &= t_{\|}(\mathbf{d}) + t_{\perp}(\mathbf{d}) \\ &= \left(1 - n^2\right) \gamma_{pp\pi} \exp\left[\lambda_2 \left(1 - \frac{|\mathbf{d}|}{c}\right)\right] + n^2 \gamma_{pp\sigma} \exp\left[\lambda_1 \left(1 - \frac{|\mathbf{d}|}{a}\right)\right]. \end{aligned} \tag{2}$$

Due to the internal twist between adjacent graphene sheets, a sufficient description of the interlayer hopping must include contributions from $pp\pi$ bonds $\gamma_{pp\pi} = -2.8$ eV as well as from $pp\sigma$ bonds $\gamma_{pp\pi} = 0.48$ eV [7]. To this end, the factor $n = \frac{\mathbf{d} \cdot \hat{\mathbf{e}}_z}{|\mathbf{d}|}$ captures the out-of plane component of the electron transfer integral. Furthermore, $\mathbf{e}_z$ is a unit vector which points perpendicular to the graphene sheets, $c = 3.364$ Å is the interlayer spacing of graphite, $a = 1.42$ Å is the distance between neighboring carbon atoms and $\lambda_1 = 3.15$ and $\lambda_2 = 7.462$ describe the exponential cutoff of the electron hopping.

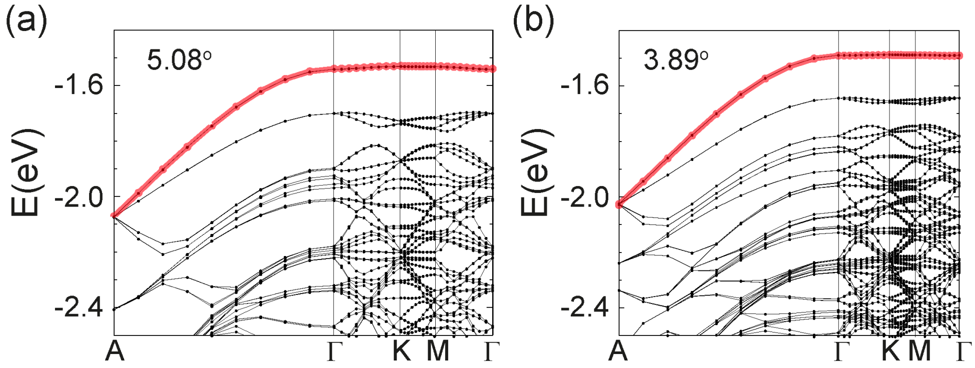

Figure S 1. Band structures of 3D twisted boron nitride with type I stacking at 5.08 degrees (a) and 3.89 degrees (b). The band width of the top valence band along the in-plane at k_z=0 decreases with twist angles, while the band width along the out-of-plane direction remains highly dispersive.

For the calculations of 3D twisted WSe_2 and boron nitride, we perform first principles calculations based on DFT as implemented in the Vienna Ab initio Simulation Package (VASP) [8] following similar methods used in previous works [9, 10]. Plane-wave basis sets are employed with an energy cutoff of 550 eV for WSe_2 and 400 eV for boron nitride. The projector augmented wave method (PAW) [11] is used to construct the pseudopotentials felt by the valence electrons. For the calculations of 3D twisted WSe_2, the exchange-correlation functionals are treated within the generalized gradient approximation (GGA) [12]. All the atoms are relaxed until the force on each atom is less than 0.01 eV/Å. Van der Waals interactions are included using the method of Tkatchenko and Scheffler [13] during the relaxation. For the calculations of 3D twisted boron nitride, the exchange-correlation functionals are treated within the local density approximation (LDA). As shown in the previous work [10], the flat bands near the top of the valence band of twisted boron nitride do not change much upon relaxation. Therefore, as the calculations for 3D twisted boron with twist angles down to 2.28 degree are very heavy, we perform these large scale calculations for 3D twisted boron nitride without relaxation.

S II. LOW-ENERGY TIGHT-BINDING MODEL FOR TWISTED BORON NITRIDE

The low-energy physics of twisted hBN (thBN) is captured by an effective three-dimensional tight-binding (TB) model that includes hopping terms between emerging charge

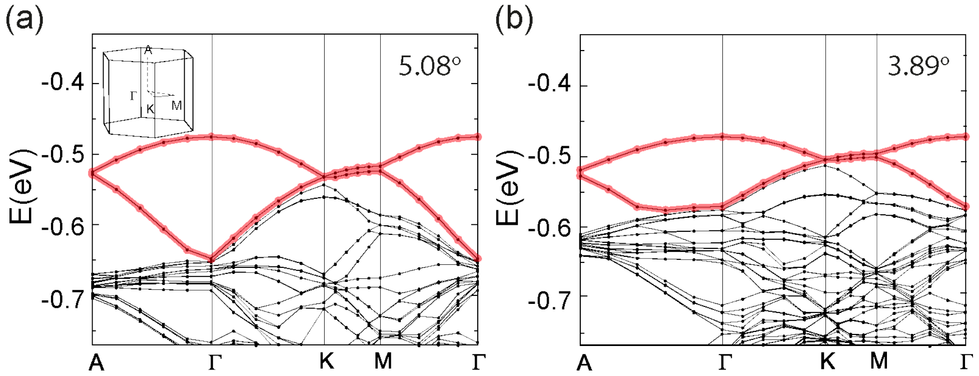

Figure S 2. Band structures of 3D twisted WSe_2 with type III stacking at 5.08 degrees (a) and 3.89 degrees (b). The band width along both the in-plane and the out-of-plane directions decreases with twist angles.

localization points at $\mathbf{Q}_1 = (\frac{1}{3}, \frac{1}{3}, \frac{1}{2})$ and $\mathbf{Q}_2 = (\frac{2}{3}, \frac{2}{3}, 0)$ in the moiré unit cell. The coordinates are given with respect to the in-plane ($\|$) and out-of plane ($\perp$) Bravais lattice vectors $\mathbf{L}_1^{\|} = (L, 0, 0)$, $\mathbf{L}_2^{\|} = R(\pi/3)\mathbf{L}_1$ and $\mathbf{L}_3^{\perp} = (0, 0, D)$. The lattice constant D is fixed, while L is twist-angle dependent and it describes the spatial extent of the moiré pattern, see table 1.

The effective structure defined by the charge accumulation points resembles AA-stacked graphene multilayers, where one of the two inequivalent sites, i.e. $\mathbf{Q}_1$, is shifted by $D/2$ in z-direction. Hence, in each of the two "effective" planes with z-coordinate 0 and $D/2$, the charge puddles form a triangular lattice with lattice constant L.

The simplest $SU(2)$ symmetric TB model that can be constructed for this configuration is a single-orbital two-band model that takes up to next-nearest neighbor intra- and interlayer hopping terms between the charge puddles into account

$$H_0 = t_1 \sum_{\langle i,j \rangle} c_i^\dagger c_j + t_2 \sum_{\langle i,j \rangle_{\|}} c_i^\dagger c_j + t_3 \sum_{\langle i,j \rangle_{\perp}} c_i^\dagger c_j. \quad (3)$$

Here, t_1 denotes the hopping amplitude between neighboring $\mathbf{Q}_1$- and $\mathbf{Q}_2$-sites , whereas t_2 and t_3 denote hopping processes between two $\mathbf{Q}_1$ ($\mathbf{Q}_2$) sites in either the same or different layers. The hopping parameters are determined by fitting the energy eigenvalues of H_0 to the flat bands of the *ab-initio* band structure of thBN. The single-particle spectrum for the

periodic system is then modeled by the following Bloch Hamiltonian

$$H_0 = \sum_{\mathbf{k}} h_{\mathbf{k}} = \sum_{\mathbf{k}} \begin{pmatrix} h_0(\mathbf{k}) & h_1(\mathbf{k}) \\ h_1^*(\mathbf{k}) & h_0(\mathbf{k}) \end{pmatrix}, \tag{4}$$

which is labeled in the order of the two charge localization points $\mathbf{Q}_1$,$\mathbf{Q}_2$. The matrix elements are obtained by a Fourier transform of the real-space hopping matrix Eq. (3) to (Bloch) momentum space

$$\begin{aligned} h_0(\mathbf{k}) &= 2t_2 \left[\cos(\mathbf{k}\cdot(\mathbf{L}_1-\mathbf{L}_2)) + \cos(\mathbf{k}\cdot\mathbf{L}_1) + \cos(\mathbf{k}\cdot\mathbf{L}_2)\right] + 2t_3\cos(\mathbf{k}\cdot\mathbf{L}_3), \\ h_1(\mathbf{k}) &= t_1 \left[1 + e^{-i\mathbf{k}\cdot\mathbf{L}_1} + e^{-i\mathbf{k}\cdot\mathbf{L}_2}\right]\left[1 + e^{-i\mathbf{k}\cdot\mathbf{L}_3}\right]. \end{aligned} \tag{5}$$

The matrix $h_{\mathbf{k}}$ can then be diagonalized in orbital space for each momentum $\mathbf{k}$ to obtain the bandstructure $\epsilon_b(\mathbf{k})$ and orbital-to-band transformation $u^b_{\mathbf{r}}(\mathbf{k})$, $b = 1..N$:

$$H_0 = \sum_{\mathbf{k},b} \epsilon_b(\mathbf{k}) \gamma^{\dagger}_{\mathbf{k},b}\gamma_{\mathbf{k},b} \qquad \text{with} \quad \gamma_{\mathbf{k},b} = u^b_{\mathbf{r}}(\mathbf{k}) c_{\mathbf{k},\mathbf{r}}. \tag{6}$$

twist angle θ	Hopping parameters (meV)			Lattice constants (Å)	
	t_1	t_2	t_3	L	D
5.08°	14.59	-4.35	0.00	28.31	12.92
3.89°	8.16	-1.56	2.08	37.00	12.92
3.15°	5.29	-1.17	1.72	45.70	12.92
2.64°	3.18	-0.68	2.04	63.10	12.92

Table 1. Hopping parameters of the effective $SU(2)$-symmetric tight-binding model for different twist angles θ according to Fig. 3 in the main text. The structure constants D and L (see Fig. 3(a)) describe the spatial extent of the moiré cell in in-plane and out-of-plane direction, respectively.

S III. FLUCTUATION EXCHANGE APPROXIMATION IN MULTI-ORBITAL SYSTEMS

A. 3D multi-orbital susceptibility

We define the free Matsubara Green's function in orbital-momentum (frequency) space as

$$g_{\mathbf{r},\mathbf{r}'}(i\omega,\mathbf{k}) = (i\omega - (H_0(\mathbf{k}))_{\mathbf{r},\mathbf{r}'})^{-1} = \sum_b u^b_{\mathbf{r}}(\mathbf{k}) g^b(i\omega,\mathbf{k}) u^{b*}_{\mathbf{r}'}(\mathbf{k}) = \underset{(b,\mathbf{k})}{\overset{\mathbf{r}\qquad\qquad\qquad\qquad\mathbf{r}'}{\xrightarrow{\qquad\qquad\qquad\qquad}}} \tag{7}$$

where $u^b_{\mathbf{r}}$ are the orbital-to-band transformations that render the unperturbed Hamiltonian H_0 and the free Green's function $g^b(i\omega,\mathbf{k}) = (i\omega - e^b(\mathbf{k}))^{-1}$ diagonal. The orbital indices $\mathbf{r} = \{\mathbf{Q}_1, \mathbf{Q}_2\}$ are restricted to the same unit cell and the momenta $\mathbf{k}$ lie in the first Brillouin zone. To this end, we define the free polarization function $\hat{\chi}_0(q) = \chi_{0_{\mathbf{r},\mathbf{r}'}}(q)$ as

$$\chi_{0_{\mathbf{r},\mathbf{r}'}}(q) = \chi_{0_{\mathbf{r},\mathbf{r}'}}(\mathbf{q}, i\omega) = \frac{1}{N\beta} \sum_{\mathbf{k},\omega'} g_{\mathbf{r},\mathbf{r}'}(i\omega',\mathbf{k}) g_{\mathbf{r}',\mathbf{r}}\left(i(\omega'+\omega), \mathbf{k}+\mathbf{q}\right). \tag{8}$$

The Matsubara summation occuring in Eq. (8) can be evaluated analytically giving the well-known Lindhard function for multi-orbital systems

$$\chi_{0_{\mathbf{r},\mathbf{r}'}}(\mathbf{q}, i\omega) = \frac{1}{N} \sum_{\mathbf{k},b,b'} \frac{n_F(\epsilon_{b'}(\mathbf{k})) - n_F(\epsilon_b(\mathbf{k}+\mathbf{q}))}{i\omega + \epsilon_{b'}(\mathbf{k}) - \epsilon_b(\mathbf{k}+\mathbf{q})} u^{b'}_{\mathbf{r}}(\mathbf{k}) u^{b'*}_{\mathbf{r}'}(\mathbf{k}) u^{b*}_{\mathbf{r}}(\mathbf{k}+\mathbf{q}) u^{b}_{\mathbf{r}'}(\mathbf{k}+\mathbf{q}), \tag{9}$$

where $n_F(\epsilon) = (1 + e^{\beta\epsilon})^{-1}$ is the Fermi function.

B. Random-phase approximation for multi-orbital systems

To study correlated states of matter in thBN that arise due to the presence of electron-electron interaction, we employ a repulsive Hubbard term for electrons with opposite spin $\sigma \in \{-1, 1\}$ with $\overline{\sigma} = -\sigma$ residing on site $\mathbf{r}$ in moiré supercell $\mathbf{R}$

$$V = \frac{1}{2} \sum_{\mathbf{R},\mathbf{r}_i,\sigma} U n_{\mathbf{R},\mathbf{r}_i,\sigma} n_{\mathbf{R},\mathbf{r}_i,\overline{\sigma}} = \frac{1}{2N} \sum_{\mathbf{k},\mathbf{k}',\mathbf{q}} \sum_{\mathbf{r},\sigma} U c^{\dagger\mathbf{r}}_{\mathbf{k},\sigma} c^{\dagger\mathbf{r}}_{\mathbf{k}',\overline{\sigma}} c^{\mathbf{r}}_{\mathbf{k}'-\mathbf{q},\overline{\sigma}} c^{\mathbf{r}}_{\mathbf{k}+\mathbf{q},\sigma} \tag{10}$$

Here, the occupation number operator is defined as $n_{\mathbf{R},\mathbf{r}_i,\sigma} = c^\dagger_{\mathbf{R},\mathbf{r}_i,\sigma} c_{\mathbf{R},\mathbf{r}_i,\sigma}$. We calculate the renormalized interactions within the random-phase approximation (RPA) to analyze the electronic instabilities mediated by spin-fluctuation exchange between electrons to high order in the bare coupling U. Admittedly, this approach is biased as it does not capture the interwind fluctuations in different two-particle scattering channels, which would require the use of unbiased fRG techniques.

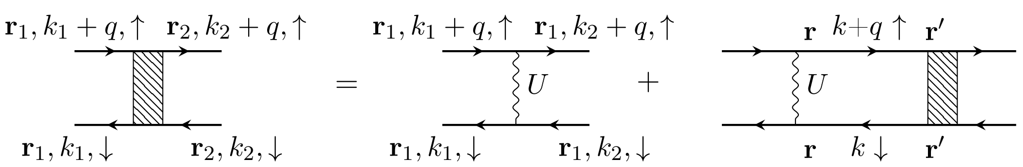

(11)

The renormalized interaction in RPA approximation Eq. (11) is then given by $\hat{V}_{\mathrm{RPA}}(q) = U/[1 + U\hat{\chi}_0(q)]$. Magnetic instabilities can be classified according to a generalized Stoner criterion: The effective (RPA) interaction diverges, when the smallest eigenvalue λ_0 of $\hat{\chi}_0(\mathbf{q}, i\omega)$ reaches $-1/U$, marking the onset of magnetic order for all interaction strengths $U \geq U_{\mathrm{crit.}} = -1/\lambda_0$. The corresponding eigenvector $v^{(0)}(\mathbf{q}, i\omega)$ is expected to dominate the spatial structure of orbital magnetisation.

C. Pairing Symmetry

We may write the general particle-particle scattering vertex between electrons with opposite momenta $(\mathbf{k}_1, -\mathbf{k}_1) \to (\mathbf{k}_2, -\mathbf{k}_2)$ as

$$V = \frac{1}{2N} \sum_{s,s'} \sum_{\mathbf{r}_1,\ldots,\mathbf{r}_4} \sum_{\mathbf{k}_1,\mathbf{k}_2} \Gamma^{\mathbf{r}_1\mathbf{r}_2\to\mathbf{r}_3\mathbf{r}_4}_{\mathbf{k}_1,-\mathbf{k}_1\to\mathbf{k}_2,-\mathbf{k}_2} c^{\dagger\mathbf{r}_3}_{\mathbf{k}_2 s} c^{\dagger\mathbf{r}_4}_{-\mathbf{k}_2 s'} c^{\;\mathbf{r}_2}_{-\mathbf{k}_1 s'} c^{\;\mathbf{r}_1}_{\mathbf{k}_1 s} =$$

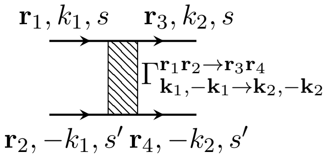

(12)

For interaction values $U < U_{\mathrm{crit}}$ the magnetic instabilities prescribed by the RPA analysis might not be strong enough to actually occur. In this paramagnetic regime, spin and charge fluctuations contained in the transverse and longitudinal spin channel can give rise to an effective interaction between electrons that may lead to the formation of Cooper pairs. The

diagrams can be separated into spin-singlet and spin-triplet contributions, depending on whether pairing same/opposite spins, i.e. $s \neq s'$ (singlet) or $s = s'$ (triplet). In general, we may separate the dependence of the gap parameter on momentum, spatial and spin degrees of freedom

$$\Delta^{\mathbf{r}_1\mathbf{r}_2}_{\mathbf{k}s_1s_2} = f(\mathbf{k}, \mathbf{r}_1, \mathbf{r}_2)\chi(s_1, s_2). \tag{13}$$

Since for spin singlet gaps the spin function $\chi(s_1, s_2)$ is antisymmetric under exchange of indices, i.e. $\chi(s_1, s_2) = -\chi(s_2, s_1)$, the spatial and momentum dependence must be symmetric in order to fulfill the Pauli principle. For spin triplet gaps we hence require $f(\mathbf{k}, \mathbf{r}_1, \mathbf{r}_2) = -f(-\mathbf{k}, \mathbf{r}_2, \mathbf{r}_1)$. Since the system is assumed to be paramagnetic, pairing same/opposite spins yields the same result after explicitly symmetrizing/anti-symmetrizing the interaction vertex in orbital-momentum space.

Restricting the pairing to Kramer's degenerate pairs $(\mathbf{k}_1, \uparrow)$ and $(-\mathbf{k}_1, \downarrow)$, the particle-particle scattering vertex in FLEX approximation is given by transverse (t) and longitudinal (l) spin fluctuations. For simplicity, we will use the abbreviation $\Gamma^{\mathbf{r}_1\mathbf{r}_2\to\mathbf{r}_3\mathbf{r}_4}_{\mathbf{k}_1,-\mathbf{k}_1\to\mathbf{k}_2,-\mathbf{k}_2} = \Gamma^{\mathbf{r}_1,\mathbf{r}_2}_{\mathbf{k}_1,\mathbf{k}_2}$ in the following. The diagrams contributing to these spin channels are shown below.

The effective spin-mediated interaction in the opposite spin channel thus becomes

$$\Gamma^{\mathbf{r}_1\mathbf{r}_2\to\mathbf{r}_3\mathbf{r}_4}_{\mathbf{k}_1,-\mathbf{k}_1\to\mathbf{k}_2,-\mathbf{k}_2} = \delta_{\mathbf{r}_1,\mathbf{r}_3}\delta_{\mathbf{r}_2,\mathbf{r}_4}\left[\hat{U} + \frac{U^3\hat{\chi}_0^2(q_l)}{1 - U^2\hat{\chi}_0^2(q_l)}\right] + \delta_{\mathbf{r}_1,\mathbf{r}_4}\delta_{\mathbf{r}_2,\mathbf{r}_3}\left[-\frac{U^2\hat{\chi}_0(q_t)}{1 + U\hat{\chi}_0(q_t)}\right] \tag{14}$$

The spin-dependence of the susceptibilities occuring in the diagrammatic expansion above can be neglected due to the emergent $SU(2)$ symmetry in the paramagnetic phase. To obtain the effective interaction in the singlet (s) and triplet (t) channel, we symmetrize/anti-symmetrize the interaction vertex, i.e.

$$\Gamma^{\mathrm{s/t}} = \frac{1}{2}$$

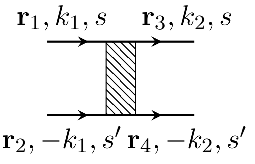

$$+\,\sigma$$

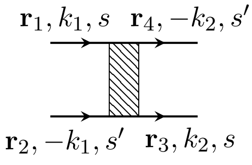

(15)

D. Linearized Gap Equation

Assuming that spin- and charge fluctuation provide the superconducting glue in the system, we confine our considerations to the vicinity of the Fermi surface and only treat

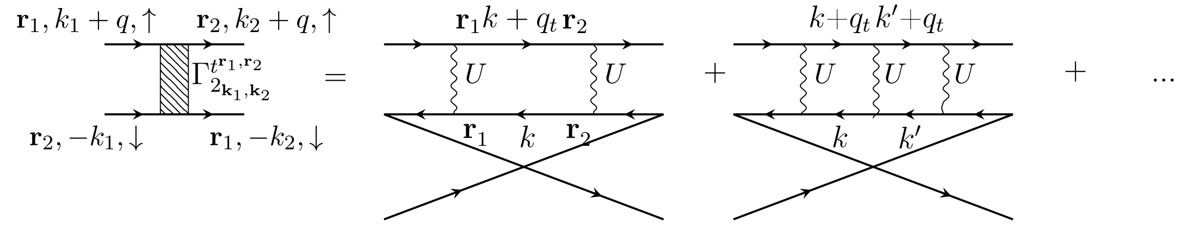

(a) Diagrams contributing to the transverse spin-fluctuation mediated pairing interaction $\Gamma^{t\,\mathbf{r}_1,\mathbf{r}_2}_{2\,\mathbf{k}_1,\mathbf{k}_2}$. The momentum transfer occurring in the polarization function in RPA is given by $q_t = k_1 + k_2$ due to momentum conservation.

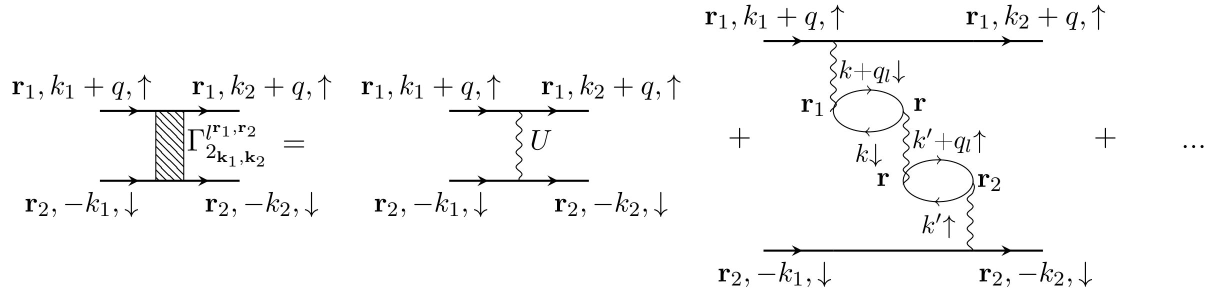

(b) Diagrams contributing to the longitudinal spin-fluctuation mediated pairing interaction $\Gamma^{l\,\mathbf{r}_1,\mathbf{r}_2}_{2\,\mathbf{k}_1,\mathbf{k}_2}$. The momentum transfer occurring in the polarization function in RPA is given by $q_l = k_1 - k_2$ due to momentum conservation. Only an even number of particle-hole bubbles is allowed in the diagrammatic expansion in order to preserve the spin in the upper and lower leg of the pairing interaction. The diagrams that are resummed in the longitudinal channel are connected to the particle-hole susceptibility describing screening effects of the bare Coulomb interaction.

scattering processes of a Cooper pair from state $(\mathbf{k}, -\mathbf{k})$ on fermi surface C_b to the state $(\mathbf{k}', -\mathbf{k}')$ on fermi surface $C_{b'}$. To this end, we project the pairing vertex Eq. (14) from orbital to band space and only take intra-band scattering into account

$$\Gamma^{bb'}_{s/t}(\mathbf{k}, \mathbf{k}') = \mathrm{Re}\left[\sum_{\mathbf{r}_1,\mathbf{r}_2,\mathbf{r}_3,\mathbf{r}_4} \Gamma^{s/t} u^{b*}_{\mathbf{r}_1}(\mathbf{k}) u^{b*}_{\mathbf{r}_2}(-\mathbf{k}) u^{b'}_{\mathbf{r}_3}(\mathbf{k}') u^{b'}_{\mathbf{r}_4}(-\mathbf{k}')\right]. \tag{16}$$

The momenta $\mathbf{k}$ and $\mathbf{k}'$ are restricted to the various fermi surface sheets $\{C\}$, such that $\mathbf{k} \in C_b$ and $\mathbf{k}' \in C_{b'}$ with b and b' being the band indices of the fermi sheets. Neglecting the frequency dependence of Γ, we can proceed further by considering only the real part of the pairing interaction. We then solve the linearized gap equation in order to obtain strength and pairing symmetry of the superconducting order parameter, which takes the form of a

generalized eigenvalue problem

$$-\frac{1}{V_{\mathrm{BZ}}}\sum_{b'}\oint_{\mathrm{FS}_{b'}}\frac{\Gamma^{bb'}_{s/t}(\mathbf{k},\mathbf{k}')}{v^{b'}_F(\mathbf{k}')}\Delta_{b'}(\mathbf{k}')=\lambda\Delta_b(\mathbf{k}). \tag{17}$$

Here, $v^b_F(\mathbf{k})=|\nabla\epsilon_b(\mathbf{k})|$ is the Fermi velocity at $\mathbf{k}'$ in band b. The largest eigenvalue $\lambda>0$ for a given interaction kernel $\Gamma^{bb'}_{s/t}(\mathbf{k},\mathbf{k}')$, will lead to the highest transition temperature T_c and the corresponding eigenfunction $\Delta_b(\mathbf{k})$ determines the symmetry of the gap. The effective lattice model obtained from the charge accumulation points has point group D_{3h}. The symmetry of the gap can thus by classified according to the irreducible representations of D_{3h} that are listed in Table 2.

The linearized gap equation (17) only accounts for the leading pairing symmetry at the transition temperature T_c of the superconducting phase. In the case of degenerate eigenvalues (e.g. d-wave instabilities $\{d_{xz},d_{yz}\}$) belonging to a two-dimensional irreducible representation, an arbitrary linear combination might be favored for $T<T_c$. In order to find the linear combination that is preferred by the system below the transition temperature, we compute the free energy of the system

$$\begin{aligned}F=E-TS=&\frac{1}{N}\sum_{\mathbf{k},b}\left[E_b(\mathbf{k})n_F(E_b(\mathbf{k}))-\frac{|\Delta_b(\mathbf{k})|}{E_b(\mathbf{k})}\tanh\left(\frac{E_b(\mathbf{k})}{2T}\right)\right]\\&+\frac{T}{N}\sum_{\mathbf{k},b}\left[n_F(E_b(\mathbf{k}))\ln(n_F(E_b(\mathbf{k})))+n_F(-E_b(\mathbf{k}))\ln(n_F(-E_b(\mathbf{k})))\right].\end{aligned} \tag{18}$$

Here, $E_b(\mathbf{k})$ is the energy of the Bogoliubov quasi-particles resulting from diagonalization of the BdG Hamiltonian

$$H_{\mathrm{BdG}}=\sum_{\mathbf{k},b}\psi^\dagger_{b\mathbf{k}}\begin{pmatrix}\epsilon_b(\mathbf{k})-\mu & \Delta_b(\mathbf{k})\\ \Delta^\dagger_b(\mathbf{k}) & -\epsilon_b(-\mathbf{k})+\mu\end{pmatrix}\psi_{b\mathbf{k}}=\sum_{\mathbf{k},b}\psi^\dagger_{b\mathbf{k}}\left[\delta_b(\mathbf{k})\cdot\tau\right]\psi_{b\mathbf{k}}, \tag{19}$$

where $\delta_b(\mathbf{k})=(\Re[\Delta_b(\mathbf{k})],\Im[\Delta_b(\mathbf{k})],\epsilon_b(\mathbf{k})-\mu)^T$ and τ are the Pauli matrices. In the expression of the free energy Eq. (18), we only account for states at the Fermi surface as contributions from $\mathbf{k}$ points far away from the Fermi surface are negligible $\epsilon_b(\mathbf{k})\gg|\Delta_b(\mathbf{k})|$.

At the filling $\mu\approx\mu_0+5\,\mathrm{meV}$ studied in the manuscript, the leading pairing symmetry is the d-wave which belongs to a two-dimensional irreducible representation. To minimize the

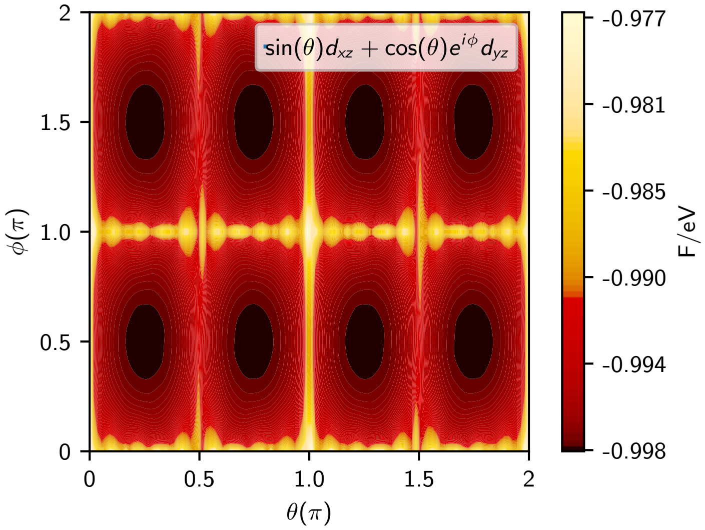

Figure S 3. Free energy of the linear combination $\Delta_b(\mathbf{k}) = \sin(\theta)d_{xz}(\mathbf{k}) + \cos(\theta)e^{i\phi}d_{yz}(\mathbf{k})$ corresponding to the leading pairing symmetry at $\mu \approx \mu_0 + 5\,\mathrm{meV}$. The system minimizes its free energy by choosing the linear combination $d_{xz}(\mathbf{k}) \pm id_{yz}(\mathbf{k})$.

free energy of the system we make the ansatz

$$\Delta_b(\mathbf{k}) = \sin(\theta)d_{xz}(\mathbf{k}) + \cos(\theta)e^{i\phi}d_{yz}(\mathbf{k}), \tag{20}$$

where the form factors are are given by $d_{xz}(\mathbf{k}) = \sin(k_x)\sin(k_z)$ and $d_{yz}(\mathbf{k}) = \sin(k_y)\sin(k_z)$. The free parameters θ and ϕ are extracted by minizing the free energy of the system Eq. (18). In Fig. 3 we show that the linear combination $\Delta^b_{\mathbf{k}} \propto [d_{xz}(\mathbf{k}) \pm id_{yz}(\mathbf{k})] = [\sin(k_x)\sin(k_z) \pm i\sin(k_y)\sin(k_z)]$ is generally preferred for the given filling.

singlet	triplet
s	p_z
$(d_{x^2-y^2}, d_{xy}) \cdot p_z$	$(d_{x^2-y^2}, d_{xy})$
(d_{xz}, d_{yz})	(p_x, p_y)
$f_{x(x^2-3y^2)} \cdot p_z$	$f_{x(x^2-3y^2)}$
$f_{y(y^2-3x^2)} \cdot p_z$	$f_{y(y^2-3x^2)}$

Table 2. Pairing symmetries for the effective lattice model of thBN separated into contributions to spin singlet and triplet channel.

REFERENCES

[1] S. Plimpton, Journal of computational physics **117**, 1 (1995).

[2] M. Angeli, D. Mandelli, A. Valli, A. Amaricci, M. Capone, E. Tosatti, and M. Fabrizio, Physical Review B **98**, 235137 (2018).

[3] D. W. Brenner, O. A. Shenderova, J. A. Harrison, S. J. Stuart, B. Ni, and S. B. Sinnott, Journal of Physics: Condensed Matter **14**, 783 (2002).

[4] A. N. Kolmogorov and V. H. Crespi, Physical Review B **71**, 235415 (2005).

[5] W. Ouyang, D. Mandelli, M. Urbakh, and O. Hod, Nano letters **18**, 6009 (2018).

[6] E. Bitzek, P. Koskinen, F. Gähler, M. Moseler, and P. Gumbsch, Physical review letters **97**, 170201 (2006).

[7] G. Trambly de Laissardière, D. Mayou, and L. Magaud, Nano Letters **10**, 804 (2010).

[8] G. Kresse and J. Hafner, Phys. Rev. B **47**, 558 (1993).

[9] L. Wang, E.-M. Shih, A. Ghiotto, L. Xian, D. A. Rhodes, C. Tan, M. Claassen, D. M. Kennes, Y. Bai, B. Kim, K. Watanabe, T. Taniguchi, X. Zhu, J. Hone, A. Rubio, A. N. Pasupathy, and C. R. Dean, Nature Materials **19**, 861 (2020).

[10] L. Xian, D. M. Kennes, N. Tancogne-Dejean, M. Altarelli, and A. Rubio, Nano Lett. **19**, 4934 (2019).

[11] P. E. Blöchl, Phys. Rev. B **50**, 17953 (1994).

[12] J. P. Perdew, K. Burke, and M. Ernzerhof, Physical review letters **77**, 3865 (1996).

[13] A. Tkatchenko and M. Scheffler, Phys. Rev. Lett. **102**, 073005 (2009).
